# The modifiers of chronic kidney disease in autosomal dominant polycystic kidney disease and the role of the endothelin-1

**Published:** 2016-01-12

**Authors:** Behzad Einollahi, Aidin Lotfiazar

**Affiliations:** ^1^Nephrology and Urology Research Center, Baqiyatallah University of Medical Sciences, Tehran, Iran

**Keywords:** Chronic kidney disease, Autosomal dominant polycystic kidney disease, Endothelin-1

**Implication for health policy/practice/research/medical education:**

This article evaluated the autosomal dominant polycystic kidney disease (ADPKD) modifiers to chronic kidney disease (CKD) such as allelic and nonallelic agents. Also, our study evaluated the role of the endotelin-1 which considered in some studies as the predictor of the ADPKD.

Autosomal dominant polycystic kidney disease (ADPKD) is one of the most common life threatening inherited kidney disease which its prevalence is approximately 0.5 to 1 in 1000 subjects of general population (1). In ADPKD there are many growing cysts, produced in both kidneys, which interact with normal renal function. The progression in the size of the cysts might take some decades such as 50% of ADPKD patients will encounter to end-stage renal disease (ESRD) requiring renal replacement therapy such as dialysis or kidney transplantation until fifth and sixth decade of life (2,3). Furthermore, some extra-renal complications may accompany with ADPKD such as cardiac and valvular lesions, intracranial aneurysms, pancreatic, hepatic, lung and spleen cysts, and diverticula which have significant effects on mortality and morbidity of the ADPKD (4,5). Similarly hypertension is one of the complications which occurs in ADPKD and hypertension can be seen prior to the renal function impairment in 60% of the patients (5). ESRD is one of the most common consequences of the ADPKD which occurs usually in elderly. ADPKD accounts for 4%-10% of the ESRD in the world (6).

The heterozygous mutations in PKD 1 (approximately 78%) and PKD 2 (approximately 13%) genes are responsible for the vast majority of the ADPKD incidence and no mutation detected (NMD) ADPKD exists in the other patients (9%) (7,8).

Both of the genetics and environmental factors considered as the modifiers of ESRD in ADPKD. It is stablished that the mutation in PKD 1 is more severe than mutation in PKD 2 as the mean age of ESRD in PKD 1 mutation is 58.1 years compared with 79.7 years in PKD 2 mutation (9). However, intrafamilial phenotypic variability in PKD 1 and PKD 2 pedigrees raised this idea that nonallelic factors, such as environmental agents and genetic modifying loci, have considerable role in this disease (10).

Previous studies exposed some agents as the modifiers for ESRD progression. PKD mutation, male gender, early onset of hypertension, frequent gross hematuria, three or more pregnancies in women mentioned as the modifier agents. Moreover, decrease in glomerular filtration rate (GFR) and increase in kidney size are as modifiers. Furthermore some laboratory factors such overt hematuria, macroalbominuria, high level of serum copeptin concerned as predicting factor for chronic kidney disease (CKD) in ADPKD (11). The vast majority of the studies considered the hypertension and its exacerbating factors as a modifier for ADPKD to CKD (12). Some studies expressed more than 40% higher incidental risk for ESRD in males (10).

Endotelin-1 (ET-1) is another agent that has been reported to exert multiple effects on renal function (13). Some studies advocate the effects of the ET-1 on the ADPKD progression to ESRD and some other studies refuse this effect (14). Annapareddy et al (15) in a recent study published in current issue of the Journal of Nephropharmacology evaluated the role of the ET-1 in ADPKD patients. Annapareddy et al studied on 108 ADPKD patients and extracted DNA sample of these patients and investigated the ET-1 tagging single nucleotide polymorphisms (tag-SNPs) in theme. They determined association between ADPKD and tag-SNPs by Cochran-Armitage trend test and they investigated this issue that ET-1 tag-SNPs is not a major modifier of CKD in ADPKD patients (15).

## Conclusion

In summary, the progression of ADPKD to CKD depends on multiple factors include allelic and non-allelic factors that can be use in prevention of the CKD in these patients and need further researches for achieve to this issue.

## Authors’ contribution

The authors contributed equally to the manuscript.

## Conflicts of interest

The authors declared no competing interests.

## Ethical considerations

Ethical issues (including plagiarism, data fabrication, double publication) have been completely observed by the authors.

## Funding/Support

None.
